# Prevalence of *Toxoplasma gondii* Antibodies and Risk Factor Investigation in Portuguese Veterinarians: A Matched Case–Control Study

**DOI:** 10.3390/pathogens11101217

**Published:** 2022-10-21

**Authors:** Daniela Almeida, Sérgio Santos-Silva, Maria Aires Pereira, Carla Santos, Cristina Mega, Catarina Coelho, Carmen Nóbrega, Fernando Esteves, Rita Cruz, Helena Vala, João R. Mesquita

**Affiliations:** 1 ICBAS-School of Medicine and Biomedical Sciences, Porto University, 4050-313 Porto, Portugal; 2 Instituto Politécnico de Viseu, Escola Superior Agrária de Viseu, Campus Politécnico, 3504-510 Viseu, Portugal; 3 Global Health and Tropical Medicine (GHTM), Instituto de Higiene e Medicina Tropical (IHMT), Universidade Nova de Lisboa (UNL), 1349-008 Lisboa, Portugal; 4 CERNAS, Instituto Politécnico de Viseu, Campus Politécnico, 3504-510 Viseu, Portugal; 5 Centre for the Research and Technology of Agro-Environmental and Biological Sciences (CITAB), University of Trás-os-Montes e Alto Douro, 5001-801 Vila Real, Portugal; 6 Epidemiology Research Unit (EPIUnit), Instituto de Saúde Pública da Universidade do Porto, 4050-313 Porto, Portugal; 7 Laboratório para a Investigação Integrativa e Translacional em Saúde Populacional (ITR), 4050-313 Porto, Portugal

**Keywords:** *Toxoplasma gondii*, IgG antibodies, veterinary practitioners, Portugal

## Abstract

(1) Background: *Toxoplasma gondii* is a widespread zoonotic agent that greatly impacts Public Health, being responsible for one of the most important parasitic zoonosis worldwide. *T. gondii* has a heteroxenous life cycle, with cats being the definitive hosts and all warm-blooded animals, including humans, being intermediate hosts. Veterinary practitioners (VP) may be at a higher risk than the general population for *T. gondii* infection, as they have direct and daily contact with many animal species. The aim of the present study was to ascertain if VP were more likely to be anti-*T. gondii* IgG seropositive than the general population, as well as to understand if age, accidents with blood-contaminated sharps (cross-blood contamination), gender, working years, and geographic regions play a role as risk factors for *T. gondii* infection. For this purpose, a case–control study using archived samples was performed. (2) Methods: A total of 350 veterinary practitioners were tested using a commercial semiquantitative enzyme immunoassay for anti-*T. gondii* IgG. From the general population, 175 anonymous volunteers (matched with cases by region, age, and gender) were studied for anti-*T. gondii* IgG. (3) Results: There was no statistical difference found between the presence of anti-*T. gondii* IgG in practitioners (26%; CI = 21.40–30.60%) and the general population (33.14%; CI = 26.17–40.12%) (*p* = 0.108). Univariate and multivariate analysis showed that only age (older groups) was found to be associated with a higher prevalence of anti-*T. gondii* IgG, with significant *p* values (*p* < 0.05) for both univariate and multivariate analysis. (4) Conclusions: To the best of our knowledge, this is the first case–control study fully focused on the prevalence of anti-*T. gondii* IgG in VP in Portugal, showing that there was no significant risk for *T. gondii* infection in veterinarians exposed daily and repeatedly to different species of animals.

## 1. Introduction

*Toxoplasma gondii* is an extensively studied agent and is considered one of the most-relevant zoonotic parasites worldwide [1,2]. *T. gondii* is a facultative heteroxenous protozoan with a complex life cycle; it may be transmitted from definitive to intermediate hosts, from intermediate to definitive hosts, as well as amongst definitive and amongst intermediate hosts [3]. Members of the *Felidae* family have been established to be the only definitive hosts of the parasite, while all warm-blooded animals (mammals and birds), including humans, can be intermediate hosts [4]. Sexual and asexual reproduction can occur in the definitive host. On the contrary, in intermediate hosts, only asexual reproduction is possible [1]. Oocysts, generated in sexual reproduction, are shed in faeces of infected felids, subsequently becoming infective after sporulation, which ensues in 1–5 days of environmental exposure [1]. The sporulated oocysts containing sporozoites can be ingested by intermediate or definitive hosts through contaminated food or water [5]. After ingestion, the sporozoites differentiate into tachyzoites, which generate a transitory parasitaemia [1]. Tachyzoites constitute the rapid proliferation form found in the acute stage of infection, while bradyzoites, originating from tachyzoites, multiply slowly, being characteristic of the chronic stage of infection, with both forms multiplying intracellularly [6]. Bradyzoites then encyst in different tissues, showing high predilection for the brain, eye, cardiac and skeletal muscles, but also for visceral organs, such as the liver, lungs and kidneys, staying latent, in a relatively dormant state in immunocompetent subjects, but may recur to active disease in immune-weakened individuals [1,7].When cysts rupture, bradyzoites are released and converted to tachyzoites that replicate, disseminate and may cause severe and life-threatening conditions, especially in the aforementioned immune-compromised individuals [8]. Both definitive and intermediate hosts, may also become infected after ingestion of these tissue cysts in raw or undercooked meat [1]. Other routes of transmission may be transplacental, organ transplantation or via unpasteurized milk, being the last two listed considered rare [2].

In humans, *T. gondii* is widespread around the world, affecting about 42% of general the population [9]. Although infection by *T. gondii* in immunocompetent hosts varies between an asymptomatic to a slightly symptomatic condition (lymphadenitis, and fever) it may cause severe encephalitis in immunosuppressed individuals, which may become fatal [10]. If acquired during pregnancy, congenital toxoplasmosis frequently induces abortion, foetal deformities, ocular (retinochoroiditis) and neurological manifestations, which may be detected at birth or later in the child’s development. Newborns may present severe neurological symptoms, such as seizures and hydrocephalus, while visual lesions, if not diagnosed at birth, may only become apparent later in life through signs of aggravated visual impairment. Both situations may potentially originate lifelong repercussions in affected children. The earlier the foetal infection in the uterus, the more severe the clinical signs of toxoplasmosis, nevertheless, early infection is less likely to occur during early pregnancy, as the placenta is less permeable for tachyzoites [1,11].

Throughout the years, it has been considered imperative not only to understand *T. gondii*’s lifecycle and how the parasite affects public and animal health but also to seek and identify the risk factors predisposing humans and animals to infection and disease. These investigations allowed for the identification of the consumption of raw/undercooked meat, a lack of hygiene in preparing vegetable and fruit, contaminated water, age, and the intensity of contact with soil and animals as important risk factors for *T. gondii* infection [2,12,13]. Occupationally exposed professions, such as veterinary practitioners (VP), slaughterhouse workers, butchers, farm producers, cheese makers, workers exposed to under-washed raw vegetables, fruits and working with soil, are commonly considered to be at a higher risk of infection by *T. gondii* due to their direct and permanent contact with potential fomites. These may be cats’ faeces, soil, fruits and vegetables contaminated with sporulated oocysts, blood contaminated with tachyzoites (parasitaemia phase), or raw meat and organs contaminated with bradyzoites [14,15,16,17,18].

The aim of this work is to ascertain a possible relationship between working as a veterinary practitioner (VP) and being seropositive for *T. gondii*, and to study if age, geographical region, gender, working years, and cross-blood contamination at work constitute occupational hazards for infection with *T. gondii*. 

## 2. Materials and Methods

### 2.1. Sampling

For this study a total of 350 archived (−80 °C) sera samples from a previous study were used [19]. These samples included Portuguese VP who attended the Annual Veterinary Meeting in January 2012 in Santa Maria da Feira, Portugal and who were working with small animals (dog/cat), livestock species (pigs, poultry, sheep/goat, horses, and bovines) and exotic animals. Retrieved participants’ information included geographical region (coastline or countryside), gender (female or male), age (19–29; 30–39; 40–49; ≥50 years old), number of working years (1–10; 11–20 and >20 years), and cross-blood contamination (accidents at work with sharp materials – scalpel, scissors, knives during practice resulting in blood contact between the VP and the animal). The participant’s age ranged from 19 to 63 years and had been working with animals for 1 year up to 40 years. Descriptive data regarding can be found in Table 1.

For the control group, archived sera samples from anonymous volunteers (*n* = 175) matched with the VP by gender and age were used in the proportion of one control to each two members of the VP group. This study was approved by the institutional review board at the University of Porto (Parecer n°18/CEUP/2011).

### 2.2. Detection of Anti-T. gondii Antibodies 

All sera samples were individually tested for the presence of anti-*T. gondii* IgG antibodies. A commercial semiquantitative enzyme immunoassay (Toxoplasma ELISA IgG G1027, Edition 2018, Vircell, Granada, Spain) was used for that purpose. This commercial ELISA contained purified *T. gondii* antigen RH (ATCC 50174). According to the manufacturer, it has a sensitivity of 98% (88–100%, CI = 95%) and a specificity of 100% (89–100%, CI = 95%). All the procedures were performed according to the manufacturer’s instructions. 

### 2.3. Data Analysis 

Data processing was firstly performed using Microsoft Office 365 Excel. After organizing all the data, the statistical analysis was performed using IBM SPSS version 28.0.0.0 statistical software. Confidence intervals were established at 95%. A chi-square with Yate’s correction test for homogeneity of proportions was used to calculate significant differences in anti-*T. gondii* IgG seroprevalence between the VP group and the control group. Binary and multinomial logistic regression analyses were carried out to determine which of the variables (region, gender, age, years of work, and cross-blood contamination) were significantly (*p* < 0.05) associated with the detection of anti-*T. gondii* IgG among VP.

## 3. Results

From the 350 VP sera samples tested, 91 were positive for the presence of anti-*T. gondii* IgG antibodies. From the 175 control samples tested, 58 were positive for the presence of anti-*T. gondii* IgG antibodies (Table 2). Seroprevalence of anti-*T. gondii* IgG in VP and control group was 26% (CI = 21.4–30.6%) and 33.1% (CI = 26.2–40.1%), respectively. Chi-square test with Yates’s correction was found to be 2.5876, showing that there is no statistical difference in anti-*T. gondii* IgG seroprevalence between VP and the control group (*p* = 0.108). 

The association between the detection of anti-*T. gondii* IgG in the VP group and the variables (region, gender, age, years of work, and work accidents) was evaluated by binomial logistic regression (univariate) and multinomial logistic regression analysis (multivariate) (Table 3). For binary regression and regarding age, being of the 30–39 and 40–49 age groups was found to be associated with the presence of anti-*T. gondii* IgG (*p* = 0.022 and *p* = 0.021, respectively). Multivariate analysis supports that age (older group; ≥50 years of age) is a risk factor for anti-*T. gondii* IgG seropositivity (*p* = 0.039). The number of working years was found to be statistically significant (*p* = 0.035) for the group with 10–20 years of work, in univariate analysis only. This significance may be due to bias caused by other variables (namely age) that, after evaluation in multivariate analysis, were corrected. None of the other variables (gender, region, years of work, and cross-blood contamination) were observed to be a significant risk factor for anti-*T. gondii* IgG seropositivity in VP group, by both univariate and multivariate analysis.

## 4. Discussion

This is the first study conducted in Portugal to determine *T. gondii* seroprevalence in VPs and the relevance of professional risk factors linked to greater *T. gondii* exposure. This serosurvey showed that overall seroprevalence of anti- *T. gondii* IgG among VPs occupationally exposed to animals, was 26%. Statistically, the lack of association between the seropositivity of both studied groups, VPs vs. the controls, shows that, in Portugal, there is no evidence of a particular occupational risk in VPs (*p* = 0.108).

These findings are in accordance with other studies evaluating the seroprevalence of anti-*T. gondii* IgG in VPs and in the general population. Recent research in India, reported an anti-*T. gondii* seroprevalence of 8.78% for VPs and a seroprevalence that varies between 5 to 80%, in the general population [18,20]. In Finland, a seroprevalence of 14.6% in VPs vs. 19.7% in general population was observed, with non-significant differences between them [17]. Likewise, in Mexico, no association was found between working as a veterinarian and being seropositive for *T. gondii* with 6% of seropositivity in VPs compared to 5.5% in the control group [21]. Similar studies from Estonia, Malaysia, and Canada reported veterinary personal seropositivity for *T. gondii* of 45.2%, 18.3%, and 14.2%, respectively, while the control group registered 55.8%, 16.3%, and 23%, showing no statistically significant differences between the surveyed populations [22,23,24]. Inversely, a recent study from central India reported a *T. gondii* seropositivity of 48.9% in VPs against 6.6% in the control group, showing an association between anti- *T. gondii* IgG and IgM and veterinary practitioners [25]. Differences in *T. gondii* seropositivity between the studies cited above can be attributed to different geographical, climatical, and socioeconomic conditions [26]. The lack of association among VPs and the controls found in most studies, may be related to the fact that veterinary practitioners are highly qualified, having a deep understanding about the parasite’s life cycle and of the safety measures necessary to avoid *T. gondii* infection while working. Moreover, VPs have contact with cats and are less likely to have contact with their faeces in which oocysts take 1–5 days to sporulate [18].

In this study, risk factors, such as gender, geographical region, cross-blood contamination, the number of working years, and age were analysed. Gender, geographical region, and cross-blood contamination were found to be non-significant in this serosurvey. One study from Finland reported that veterinarians living in the countryside had a four-fold greater risk of seropositivity then those who did not [11]. In relation to number of working years, the group with 10–20 years of experience presented, in univariate analyses, approximately twice the likelihood of seropositivity against *T. gondii* vs. the reference group (1–10 years) with a *p* = 0.035, which is not supported by multivariate analyses (*p* = 0.857). 

Age was the only variable studied having a statistically significant relevance in both uni- and multivariate analysis as a risk factor. Older professional groups, 30–39 years, 40–49 years, and more than 50 years were increasingly more likely to be seropositive against *T. gondii* than younger groups. For example, in multivariate analysis, being more than 50 years old increased 10.6 times the odds of testing positive for anti-*T. gondii* IgG (*p* = 0.039). We found congruent results with other studies concerning “age” in VPs as a risk factor [16,17,23,27]. Increase in seropositivity with age is not an unexpected finding, as the probability of contact, and therefore possible infection by *T. gondii*, grows cumulatively during a life-time of exposure to the parasite. Associated with age, a study from Malaysia found “the number of working years” as a risk factor, as older workers have been more exposed to *T. gondii*. Along with aging, more experienced VPs may reduce safety and hygiene measures during labour, leading to more opportunities for infection. Moreover, with age, there is an increased individual tendency for comorbidities and co-infection, which can result in a less effective immune system, facilitating *T. gondii* infection [23].

The variable “Age” is a risk factor identified in many serosurveys, but it cannot be considered a risk factor associated to the occupationally exposed, meaning that, most likely, there are other risk factors affecting *T. gondii* exposure, in VPs, in some studies. The type of practice could have an important role in infection as seen in other locations, such as Malaysia and Finland, where livestock animal VPs showed greater prevalence, or the lack of protective equipment use, soil contact and proximity with cats, and increased infection, such as in central India [17,23,25]. 

Nevertheless, risk factors non-associated with occupational exposure, such as non-hygienic food preparation at home (underwashed vegetables and fruits), consumption of undercooked or raw meat, preference for pork or small ruminants’ meat and ingestion of unsafe water (possibly contaminated with oocysts) must also be considered, as they have an important role in all the studied populations [2,4,28]. Therefore, each individual (occupationally exposed or not), should prevent risk behaviours by cooking meat for at least 4 min at 67 °C, eat clean vegetables and fruits (washed and disinfected), use protective equipment during gardening and litterbox cleaning, and washing hands after contact with raw meat or vegetables, for example [1.2]. 

In our study, important risk factors for the presence of anti- *T. gondii* IgG associated with occupational exposure were not evaluated, such as the type of practice (companion animal, or livestock animal) and the use, or not, of protective equipment. Non-associated occupational exposure risk factors, such as eating preferences and home hygiene were also not included.

## 5. Conclusions

In conclusion, the results from this study showed that being a VP in Portugal was not associated with a higher seropositivity for *T. gondii*. Age was found to be an important risk factor but was not found to be exclusive for VP. Other risk factors not related to professional exposure, which are known to increase the risk for *T. gondii* seropositivity, should be considered in future studies. 

## Figures and Tables

**Table 1:** Distribution of veterinary practitioners by region, gender, age, years of work, and accidents at work.

Variable	No. Total/%
Region	
Coastline	232/66.3%
Countryside	118/33.7%
Gender	
Female	252/72%
Male	98/28%
Ages	
19–29 years	187/53.4%
30–39 years	139/39.7%
40–49 years	18/5.1%
≥50 years	6/1.7%
Years of work	
1–10 years	291/83.1%
11–20 years	50/14.3%
>20 years	9/2.6%
Cross-Blood Contamination	
Yes	274/78.3%
No	76/21.7%

**Table 2:** Distribution of positive and negative anti-*T. gondii* IgG antibody results from the veterinary practitioner’s serum samples (*n* = 350) and the controls (*n* = 175).

	Positive No./% Anti-*T. gondii* IgG Antibody	Negative No./% Anti-*T. gondii* IgG Antibody	Total
VP	91/26%	256/74%	350
Controls	58/33.1%	117/66.9%	175
Total	149/28.4%	376/71.6%	525

**Table 3:** Univariate and multivariate analysis of risk factors for anti-*T. gondii* IgG seropositivity.among Portuguese veterinary practitioners.

Variable	No. Positive (%)	Univariate Analysis cOR (95% CI)/*p* Value	Multivariate Analysis aOR (95% CI)/*p* Value
**Cross-Blood Contamination**			
No	13/17.1%	Ref.	Ref.
Yes	78/28.5%	0.5(0.3–1)/0.048	0.6(0.3–1.1)/0.101
**Gender**			
Female	66/26.2%	Ref.	Ref.
Male	25/25.5%	1(0.6–1.8)/0.896	0.8(0.5–1.5)/0.567
**Age**			
19–29 years	37/19.8%	Ref.	Ref.
30–39 years	43/30.9%	1.8(1.1–3)/0.022	1.6(0.9–2.8)/0.089
40–49 years	8/44.5%	3.2(1.2–8.8)/0.021	3.8(1–14.8)/0.053
≥50 years	3/50 %	4.1(0.8–20.9)/0.094	10.6(1.1–99.4)/0.039
**Region**			
Coastline	63/27.2%	Ref.	Ref.
Countryside	28/23.7%	0.8(0.5–1.4)/0.490	0.9(0.5–1.5)/0.628
**Years of Work**			
1–10 years	69/23.7%	Ref.	Ref.
11–20 years	19/38%	2(1–3.7)/0.035	1.1(0.5–2.4)/0.857
≥20 years	3/33.3%	1.6(0.4–6.6)/0.509	0.3(0.04–2.5)/0.276

cOR: crude odds ratio, aOR: adjusted odds ratio, Ref.: variables reference level, CI: confidence interval.

## Data Availability

Not applicable.

## References

[1] Hill D.E., Dubey J., Abbott R.C., van Riper C., Enright E.A. (2014). Toxoplasmosis. Circular.

[2] Koutsoumanis K., Allende A., Alvarez-Ordóñez A., Bolton D., Bover-Cid S., Chemaly M., Davies R., de Cesare A., Herman L., Hilbert F. (2018). Public health risks associated with food-borne parasites. EFSA J..

[3] Tenter A.M., Heckeroth A.R., Weiss L.M. (2000). Toxoplasma gondii: From animals to humans. Int. J. Parasitol..

[4] Dubey J.P. (2010). Toxoplasmosis of Animals and Humans—2nd Edition—J. P. Dubey—Rou.

[5] Shapiro K., Bahia-Oliveira L., Dixon B., Dumètre A., de Wit L.A., VanWormer E., Villena I. (2019). Environmental transmission of Toxoplasma gondii: Oocysts in water, soil and food. Food Waterborne Parasitol..

[6] Attias M., Teixeira D.E., Benchimol M., Vommaro R.C., Crepaldi P.H., de Souza W. (2020). The life-cycle of Toxoplasma gondii reviewed using animations. Parasites Vectors.

[7] Christiansen C., Maus D., Hoppenz E., Murillo-León M., Hoffmann T., Scholz J., Melerowicz F., Steinfeldt T., Seeber F., Blume M. (2022). In vitro maturation of Toxoplasma gondii bradyzoites in human myotubes and their metabolomic characterization. Nat. Commun..

[8] Skariah S., McIntyre M.K., Mordue D.G. (2010). Toxoplasma gondii: Determinants of tachyzoite to bradyzoite conversion. Parasitol. Res..

[9] Rahmanian V., Rahmanian K., Jahromi A.S., Bokaie S. (2020). Seroprevalence of toxoplasma gondii infection: An umbrella review of updated systematic reviews and meta-analyses. J. Fam. Med. Prim. Care.

[10] Weiss L.M., Dubey J.P. (2009). Toxoplasmosis: A history of clinical observations. Int. J. Parasitol..

[11] Peyron F., L’ollivier C., Mandelbrot L., Wallon M., Piarroux R., Kieffer F., Hadjadj E., Paris L., Garcia -Meric P. (2019). Maternal and Congenital Toxoplasmosis: Diagnosis and Treatment Recommendations of a French Multidisciplinary Working Group. Pathogens.

[12] Jones J.L., Dargelas V., Roberts J., Press C., Remington J.S., Montoya J.G. (2009). Risk factors for Toxoplasma gondii infection in the United States. Clin. Infect. Dis..

[13] Fernandes M.M., Ribeiro M.M. (2020). Incidence and Risk Factors of *Toxoplasma gondii* in Workers that Occupationally Handle Animals: A Systematic Review. Stud. Syst. Decis. Control..

[14] Alvarado-Esquivel C., Liesenfeld O., Márquez-Conde J.A., Estrada-Martínez S., Dubey J.P. (2010). Seroepidemiology of infection with toxoplasma gondii in workers occupationally exposed to water, sewage, and soil in Durango, Mexico. J. Parasitol..

[15] Alvarado-Esquivel C., Estrada-Martínez S., Liesenfeld O. (2011). Toxoplasma gondii infection in workers occupationally exposed to unwashed raw fruits and vegetables: A case control seroprevalence study. Parasites Vectors.

[16] Wójcik-Fatla A., Sroka J., Zając V., Zwoliński J., Sawczyn-Domańska A., Kloc A., Bilska-Zając E., Chmura R., Dutkiewicz J. (2018). Study on Toxoplasma gondii, Leptospira spp., Coxiella burnetii, and Echinococcus granulosus infection in veterinarians from Poland. J. Vet. Res..

[17] Siponen A.M., Kinnunen P.M., Koort J., Kallio-Kokko H., Vapalahti O., Virtala A.M., Jokelainen P. (2019). Toxoplasma gondii seroprevalence in veterinarians in Finland: Older age, living in the countryside, tasting beef during cooking and not doing small animal practice associated with seropositivity. Zoonoses Public Health.

[18] Thakur R., Sharma R., Aulakh R.S., Gill JP S., Singh B.B. (2022). Seroprevalence and risk factor investigation for the exposure of Toxoplasma gondii among veterinary personnel in Punjab, India. Comp. Immunol. Microbiol. Infect. Dis..

[19] Mesquita J.R., Costantini V.P., Cannon J.L., Lin S.C., Nascimento M.S., Vinjé J. (2013). Presence of antibodies against genogroup VI norovirus in humans. Virol. J..

[20] Khan M.-U., Rashid I., Akbar H., Islam S., Riaz F., Nabi H., Ashraf K., Singla L.D. (2017). Seroprevalence of Toxoplasma gondii in South Asian countries. Rev. Sci. Tech. Off. Int. Epiz..

[21] Alvarado-Esquivel C., Pacheco-Vega S.J., Hernández-Tinoco J., Saldaña-Simental D.E., Sánchez-Anguiano L.F., Salcedo-Jáquez M., Ramos-Nevárez A., Liesenfeld O., Márquez-Conde J Á., Cerrillo-Soto S.M. (2014). Lack of association between Toxoplasma gondii infection and occupational exposure to animals. Eur. J. Microbiol. Immunol..

[22] Shuhaiber S., Koren G., Boskovic R., Einarson T.R., Soldin O.P., Einarson A. (2003). Seroprevalence of Toxoplasma gondii infection among veterinary staff in Ontario, Canada (2002): Implications for teratogenic risk. BMC Infect. Dis..

[23] Brandon-Mong G.J., Nurul Asma Anati CM S., Sharma R., Andiappan H., Tan C., Lim YA L., Nissapatorn V. (2015). Seroepidemiology of toxoplasmosis among people having close contact with animals. Front. Immunol..

[24] Lassen B., Janson M., Viltrop A., Neare K., Hütt P., Golovljova I., Tummeleht L., Jokelainen P. (2016). Serological Evidence of Exposure to Globally Relevant Zoonotic Parasites in the Estonian Population. PLoS ONE.

[25] Deshmukh A.S., Hebbar B.K., Mitra P., Shinde S., Chaudhari S., Barbuddhe S.B. (2021). Seroprevalence and risk factors of Toxoplasma gondii infection among veterinary personnel and abattoir workers in Central India. Parasitol. Int..

[26] Al-Malki E.S. (2021). Toxoplasmosis: Stages of the protozoan life cycle and risk assessment in humans and animals for an enhanced awareness and an improved socio-economic status. Saudi J. Biol. Sci..

[27] Rosypal A.C., Houk A.E., Zajac A.M., Lindsay D.S. (2015). Prevalence of IgG Antibodies to T oxoplasma gondii in Veterinary and Undergraduate Students at V irginia Tech, B lacksburg, V irginia. Zoonoses Public Health.

[28] OIE (2019). Toxoplasmosis. OIE Terrestrial Manual.

